# Integrated Insights into Drought Tolerance Mechanism of the Autotetraploid from *Gossypium herbaceum* by Transcriptome and Physiological Analyses

**DOI:** 10.3390/genes17040470

**Published:** 2026-04-17

**Authors:** Lili Feng, Lexiang Wang, Jiamin Li, Xianglong Li, Erhua Rong, Yuxiang Wu

**Affiliations:** College of Agriculture, Shanxi Agricultural University, Jinzhong 030801, China; 20232173@stu.sxau.edu.cn (L.F.); 202430222@stu.sxau.edu.cn (L.W.); 20233089@stu.sxau.edu.cn (J.L.); 202420123@stu.sxau.edu.cn (X.L.); sxaur2h@126.com (E.R.)

**Keywords:** *Gossypium herbaceum*, autotetraploid, drought stress, transcriptome analysis

## Abstract

Background: Information on the autopolyploid of *Gossypium herbaceum* remains limited until now. Previously, the autotetraploid of *G. herbaceum* was successfully generated via colchicine-induced chromosome doubling from the diploid cultivar ‘Hongxing’ in our lab. Methods: To investigate the drought stress response mechanism of this tetraploid, the autotetraploid S4 was used as the experimental material. The plants were subjected to drought stress during the flowering stage, followed by measurements of physiological and biochemical indicators and transcriptomic sequencing analysis. Results: Under drought stress, MDA content increased, and cell membranes sustained oxidative damage. Photosynthetic parameters, such as net photosynthetic rate (*Pn*), were significantly suppressed, while the activity of osmotic regulators and key antioxidant enzymes increased significantly. After rehydration, all of the above physiological indicators showed varying degrees of recovery. Transcriptome analysis revealed that, when comparing the treatment group with the control group, a total of 5530 differentially expressed genes (DEGs) were identified, with 2714 up-regulated and 2816 down-regulated. Furthermore, this study investigated the drought resistance mechanism involving the interaction between the MAPK signaling pathway and other metabolic pathways in the autotetraploid. Nine drought-resistant genes, including *MAPK3*, *bHLH47*, *GaRbohD*, *RIBA1*, *PIP1-3*, *RCA1*, *RbohD*, *CYP707A* and *HSP70*, were selected and analyzed using real-time quantitative PCR; the results were generally consistent with the transcriptomic data. Conclusions: These findings substantially enhance our understanding of the molecular mechanisms underlying drought responses in autotetraploids. This novel autotetraploid genotype expands the available cotton germplasm resources and is expected to hold significant value for research on polyploidy evolution.

## 1. Introduction

The cotton genus *Gossypium* is a useful model for the study of polyploid genome evolution, which contains 52 species comprising 45 diploids (2n = 2X = 26) and seven allopolyploids (2n = 4X = 52) [1]. There are four independently domesticated cultivated cottons, which include the two A-genome species *G. herbaceum* (A1) and *G. arboreum* (A2) and the two allopolyploids *G. hirsutum* (AD1, Upland cotton) and *G. barbadense* (AD2, Pima cotton). A translocation between chromosome 1 and chromosome 2 that differentiates A2 from all other genomes was also detected and is congruent with the inversion noted by comparative genomics between A1 and A2 [2]. Early on, Gerstel tested via cytogenetic studies that A1 was more closely related to the A-genome in the allopolyploids than A2 [3]. The significance of polyploidy in plants has long been recognized, with respect to both species diversification and implications for genetic improvement during plant breeding [4]. Duplicated genes in polyploid organisms, or homoeologs, are coordinated in several ways, mediating gene dosage effects and gene balance [5,6,7,8]. Gene expression is also governed at the level of transcription by interactions between cis- and trans-acting regulatory elements [9,10,11]. Insights derived from comparative genomics are emerging at an ever-increasing pace, enabled by genome assemblies as well as transcriptomic and other omics resources [12]. Compared with diploid cotton, tetraploid cotton has a clear polyploidy advantage with high yield, strong adaptability to the environment, and superior fiber quality [13,14].

Drought stress is one of the most significant abiotic stress factors limiting cotton growth, yield, and fiber quality. In recent years, researchers have conducted systematic analyses of the mechanisms underlying cotton’s response to drought using high-throughput sequencing technologies. At the diploid level, Ranjan et al. [15] found that transcription factors such as AP2/ERF, MYB, WRKY, and LEA were significantly up-regulated in drought-tolerant genotypes, revealing the molecular basis by which diploid cotton materials respond to drought through the regulation of root architecture and stress signaling pathways. Ranjan et al. [16] analyzed the transcriptomes of cotton leaves and roots under drought stress and identified a large number of drought-responsive genes. Yang et al. [17] performed transcriptomic sequencing on leaves from the diploid and allopolyploid offspring of cotton and found that antioxidant enzyme activity in the allopolyploid tetraploids was significantly higher than in the diploids. They also identified that most stress-related genes were up-regulated, indicating that polyploid plants outperform diploids in terms of stress tolerance, growth vigor, and metabolism. Although numerous studies have reported on cotton drought tolerance, these have primarily focused on diploid and allopolyploid species. The molecular mechanisms underlying drought tolerance in the autotetraploid of *G. herbaceum* remain unclear.

Here, this study utilized the stable S4 generation of a *G. herbaceum* autotetraploid line, which was artificially induced and obtained through four consecutive generations of self-pollination. After years of preservation in the greenhouse during winter, it was found that the autotetraploid of *G. herbaceum* has superior advantages in cold and drought resistance. In order to further screen the genes related to drought resistance in the autotetraploid, transcriptome sequencing analysis was used for the autotetraploid after drought stress. Our study also aims to provide new insights into the drought tolerance mechanism and novel materials for next drought resistant breeding in cotton.

## 2. Materials and Methods

### 2.1. Plant Materials and Growth Conditions

The experiment was conducted in the greenhouse of Shanxi Agricultural University. This experiment utilized the S4 generation (artificially autopolyploid lines in the fourth generation of inbred stable lines) of autotetraploid (A1A1A1A1, 2n = 4x = 52) as the research subject. This line was derived from the laboratory’s earlier work, in which colchicine-induced chromosome doubling was performed on the diploid *G. herbaceum* ‘Hongxing’. The chromosome overlap was successfully obtained by the root tip squashing technique and flow cytometry ploidy identification of *G. herbaceum* [18]. Plump autotetraploid seeds were selected and soaked in water for 24 h, then transferred to a constant-temperature incubator (28 °C, 1500 lux, 90% humidity, 8 h light/16 h dark) for germination. Upon the emergence of true leaves, seedlings were transferred to nutrient pots in the greenhouse for cultivation. When the root length reached 3~5 cm, plants were transplanted into big flower pots (height 18 cm, bottom diameter: 31 cm, external diameter 24 cm). The potting substrate is a blend of nutrient-rich soil and vermiculite, ensuring optimal water retention and aeration. Plants were cultivated within a controlled climate greenhouse, maintained at a day/night temperature of 28 ± 2 °C/22 ± 2 °C, with relative humidity sustained at 60–70%. The photoperiod comprises 14 h of light followed by 10 h of darkness, under a light intensity of approximately 600 μmol·m^−2^·s^−1^.

### 2.2. Design of Experiments

This experiment initially employed a completely randomized design, with all experimental plants randomly distributed throughout the greenhouse. During the experiment, fans were operated to maintain consistent temperatures. To minimize the potential impact of environmental gradients within the greenhouse, a checkerboard-style staggered layout was also adopted during placement to ensure environmental effects were uniformly confounded across treatments. Three treatments were set up with three biological replicates. During data collection, each experimental unit (each potted plant) underwent three instrumental measurements. The arithmetic mean of these measurements was used for subsequent statistical analysis to reduce measurement error. The experimental material was grown in a greenhouse and selected in potted autotetraploid S4. All testing plants were at the flowering stage and exhibited uniform growth. Before the experiment, all plants received routine water and fertilizer management to ensure a consistent baseline of physiological conditions. Be sure to begin water restriction when the plants enter full bloom (when the first flower has opened on 50% of the plants). Quantify drought severity using a morphological wilting scale: 1 = no wilting; 2 = slight curling of leaf margins; 3 = slight drooping of leaves; 4 = noticeable drooping of leaves/moderate yellowing; 5 = severe wilting/desiccation. The experiment consisted of three treatment groups: (i) control group (CK): watered every two days to maintain soil relative water content (SRWC) at 75–80%; (ii) drought stress group (CX): subjected to natural drought by ceasing watering after initial thorough irrigation; and (iii) re-watering group (RW): immediately following the 8-day drought stress, restoring soil relative moisture to 75–80% and maintaining this level for 8 days.

The severity of drought stress was comprehensively assessed by continuously monitoring morphological indicators (leaf wilting severity, yellowing, and drooping) and soil surface moisture content. Sampling is conducted when most plants exhibit stage 4 morphological wilting symptoms (which are typically reached on the 8th day after water supply is stopped), signifying the stress endpoint. Samples were taken between 10:00 and 11:00 AM on the final day of treatment. Each treatment included three biological replicates. Three to six intact functional leaves were selected from the upper to lower parts of each plant, rapidly frozen in liquid nitrogen, and transferred to a −80 °C ultra-low temperature freezer for storage. These samples were used for total RNA extraction and transcriptome sequencing and sent to Beijing Biomics Biotech Co., Ltd. (Beijing, China). Leaf samples for physiological measurements were collected simultaneously, similarly frozen and stored at −80 °C. Additionally, after 8 days of rewatering treatment, leaf samples from the rewatered group were collected during the same time period as described above for physiological parameter measurements.

### 2.3. Determination of Photosynthetic and Physiological Indices

A CI-340 portable photosynthesis system was employed to determine the net photosynthetic rate (*Pn*), intercellular CO_2_ concentration (*Ci*), transpiration rate (*Tr*), and stomatal conductance (*Gs*). Relative chlorophyll content (SPAD) was measured using a SPAD-502 handheld chlorophyll meter. Plant height was measured using a tape measure. Antioxidant enzyme activities and osmotic regulator content were determined using kits from Beijing Boxbio Science & Technology Co., Ltd. (Beijing, China). Superoxide dismutase (SOD) was measured by the WST-1 assay, peroxidase (POD) assayed by measuring the rate of brown product formation per unit of time, catalase (CAT) measured using the ammonium molybdate colorimetric method, malondialdehyde (MDA) determined via the thiobarbituric acid (TBA) assay, proline (Pro) quantified by the acidic ninhydrin colorimetric method, soluble sugar content (SS) determined using the phenol-sulfuric acid method, and reduced glutathione (GSH) measured based on the DTNB assay.

### 2.4. RNA Extraction and Transcriptome Sequencing

RNA integrity was assessed using the RNA Nano 6000 Assay Kit of the Bioanalyzer 2100 system (Agilent Technologies, Santa Clara, CA, USA), and RNA Integrity Number (RIN). All samples met requirements for subsequent experiments. mRNA was purified from total RNA using poly-T oligo-attached magnetic beads, followed by end repair and adenylation. Fragments of approximately 150~200 bp were selected using the AMPure XP system (Beckman Coulter, Beverly, MA, USA). At last, PCR products were purified (AMPure XP system), and library quality was assessed on the Agilent Bioanalyzer 2100 system. The library preparations were sequenced on an Illumina Hiseq2500/X platform, and 125/150 bp paired-end reads were generated.

### 2.5. Transcriptomic Analysis

Raw data (raw reads) of fastq format were first processed through in-house Perl scripts. Raw reads were cleaned by removing those containing adapter contamination and low-quality reads (a quality score of Q < 20). A reference genome index was constructed using Bowtie v2.2.3. The reference genome selected for this study was the *G. arboreum* reference genome [19], as *G. herbaceum* and *G. arboreum* are sister species, both belonging to the A genome and exhibiting high genomic synteny. TopHat v2.0.12 [20] was used to align the cleaned paired-end reads to this reference genome, achieving an alignment rate of 89.76–92.76%. Considering that heterologous mapping may introduce bias, we set stringent alignment parameters (maximum mismatches ≤ 5, retaining only uniquely mapped reads) and validated key candidate genes via qRT-PCR. StringTie was used for transcript assembly and quantification of gene expression levels (FPKM). Cuffquant and cuffnorm (v2.2.1) were used to calculate the FPKM values of genes in each sample [21]. The DESeq2 R package was used to carry out differential expression analysis of pairs of treatments. Genes with an adjusted |Fold Change| ≥ 2 and FDR < 0.01 found by DESeq2 were assigned as differentially expressed. GO and KEGG pathway enrichment analysis on DEGs was performed using the cluster Profiler R package [22]. Future studies could use the published reference genome of cotton to conduct independent validation, thereby further confirming the reliability of the findings in this study [2].

### 2.6. qRT-PCR

Real-time quantitative PCR was performed using the ABI 2720 PCR system. Three biological replicates were used. RNA was extracted using the TianEnze CAT#:71203-50 Plant RNA Extraction Kit. The required RNA purity was an A260/A280 ratio of 1.9–2.1, and integrity was confirmed by agarose gel electrophoresis. The primer sequences are shown in Table 1, and specificity was confirmed by melting curve analysis (single peak). The relative expression level of each gene was calculated by the 2^−ΔΔCt^, and *GhUBQ7* was used as an internal control for other genes [23].

### 2.7. Statistical Analysis

Data processing was performed using the R package and Microsoft Excel 2018. The heatmaps were drawn using TBtools II. All data were statistically analyzed using GraphPad Prism version 10.1.2 software. Before conducting the analysis, we first test the data for normality and homogeneity of variance. If the data meet the prerequisites for parametric testing, we use one-way analysis of variance (ANOVA). When ANOVA indicated a significant main effect or interaction (*p* < 0.05), Tukey’s HSD test was used for multiple comparisons to control the family-wise error rate. Multiple comparison results are indicated by asterisks (*) in figures and tables. ns denotes no significant difference (*p* > 0.05). All data are presented as mean ± standard deviation. For the PPI network, we first used OrthoVenn2 to map the selected pathway genes to homologous genes in *Arabidopsis thaliana*. Next, orthologs were identified by querying the STRING database, and the network was visualized using Cytoscape.

## 3. Results

### 3.1. Morphological Changes in Autotetraploid Under Drought Stress

**Figure 1:**
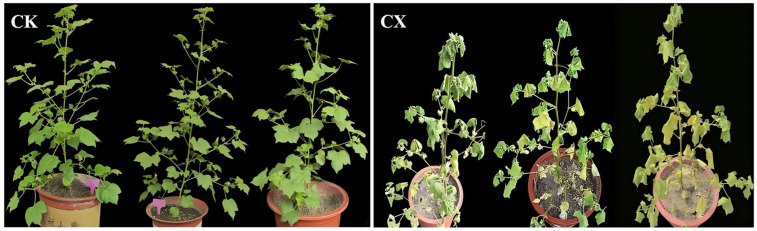
Morphological changes in autotetraploid under drought stress; CK: normal watering management, CX: subjected to natural drought by ceasing watering after initial thorough irrigation.

**Figure 2:**
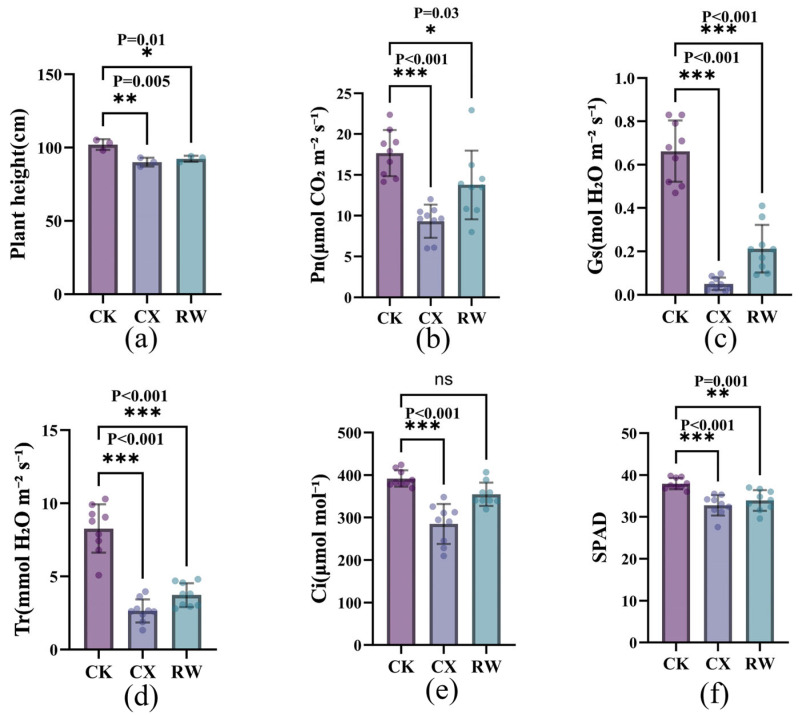
Effects of drought stress and re-watering on key physiological traits of *G. herbaceum* autotetraploid. (**a**) Plant height; (**b**) *Pn*; (**c**) *Gs*; (**d**) *Tr*; (**e**) *Ci*; (**f**) SPAD. * represents significant difference among the different treatments in the same varieties at *p* < 0.05, ** represents a significant difference among the different treatments in the same varieties at *p* < 0.01, *** represents a significant difference among the different treatments in the same varieties at *p* < 0.001, and ns represents no significant change. Error bars represent standard deviation (SD).

Over nearly a decade of greenhouse overwintering observations, the autotetraploid exhibited cold and drought resistance. Here, flowering plants of the S4 generation were selected for drought stress treatment. The results indicated significant phenotypic alterations after 8 days of drought treatment (Figure 1). Compared with the control group (CK), the drought stress group (CX) showed a 11.8% reduction in plant height (Figure 2a). Leaves of the treated plants exhibited marginal softening and drooping, with some leaves undergoing chlorosis (yellowing) and drying. Additionally, the apical growth points appeared sunken with shortened internodes, and the plants displayed pronounced wilting symptoms, indicating severe damage. On the afternoon following the end of drought treatment, the stressed plants were re-watered to saturation. All plants survived after being watered, but one plant in the treatment group failed to produce pods, as observed during the late flowering stage; this may have been due to stress.

### 3.2. Changes in Cell Membrane Permeability and Chlorophyll Content Under Drought Stress

As shown in Figure 2b–f, compared with CK, *Gs* in CX decreased by approximately 92.4% (from 0.66 to 0.05 mol H_2_O·m^−2^·s^−1^), while *Ci* significantly reduced by 26.9% (*p* < 0.001), and *Pn* declined by 47.3%, and *Tr* decreased by 67.9%. After RW, *Ci* essentially returned to control levels (354.7 μmol·mol^−1^), while *Gs* only recovered to 31.8% of CK, remaining significantly suppressed. Notably, the recovery of *Pn* reached 78% of the control level, significantly higher than that of *Gs*. Furthermore, SPAD significantly declined under drought stress (*p* < 0.001) and did not fully recover after re-watering. In summary, drought stress significantly inhibited stomatal conductance and photosynthesis in the autotetraploid. Some physiological functions exhibited compensatory recovery upon RW, with the stress group inducing photosynthetic suppression primarily driven by stomatal limitation.

### 3.3. Effects of Drought Stress on Leaf Osmotic Adjustment Substances

**Figure 3:**
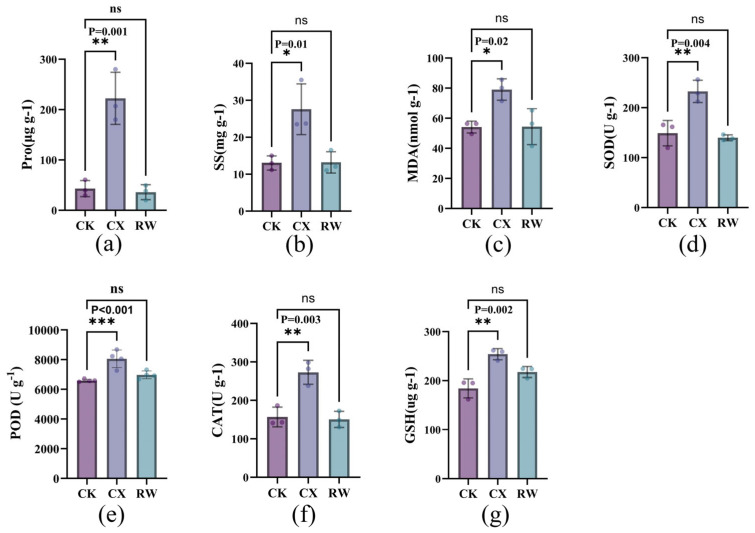
Comparison of physiological parameters in autotetraploid *G. herbaceum* under control and drought treatments, including (**a**) Pro; (**b**) SS content; (**c**) MDA content; (**d**) SOD activity; (**e**) POD activity; (**f**) CAT; (**g**) GSH. Error bars represent the standard deviation (SD) of three biological replicates. * represents significant difference among the different treatments in the same varieties at *p* < 0.05, ** represents a significant difference among the different treatments in the same varieties at *p* < 0.01, *** represents a significant difference among the different treatments in the same varieties at *p* < 0.001 and ns represents no significant change.

Drought stress significantly induced the accumulation of osmoregulatory substances in the leaves of autotetraploid (Figure 3a,b). Pro content surged to 5.2-fold (*p* = 0.001), rapidly declining to levels below the control after rehydration. SS content increased from 13.04 mg/g to 27.58 mg/g. Following rehydration, SS content recovered to control levels, exhibiting a trend consistent with Pro. These findings collectively demonstrate cotton’s capacity for efficient mobilization of small-molecule solutes for osmotic regulation under drought conditions.

### 3.4. Effects of Drought Stress on Malondialdehyde Content and Antioxidant Enzyme Activities

Drought stress significantly triggered the antioxidant defense mechanisms in cotton, and various indicators exhibited distinct recovery patterns after rewatering (Figure 3c–g). MDA content rose from 54.10 nmol/g to 79.07 nmol/g (*p* = 0.02). SOD activity significantly increased (*p* = 0.004), rising from 148.98 U/g to 232.58 U/g, while CAT activity increased from 156.84 U/g to 272.93 U/g. POD activity was also significantly elevated. The significant enhancement of SOD, POD, and CAT activities constituted an efficient ROS scavenging system, confirming that the potent antioxidant enzymatic defense capacity of cotton was successfully activated to cope with drought-induced oxidative stress. After rehydration, SOD, POD, and CAT activities all returned to control levels, with SOD and CAT showing a slight decline.

GSH is a crucial endogenous antioxidant that not only directly scavenges ROS but also serves as a substrate for glutathione peroxidase, thereby participating in the degradation of H_2_O_2_ and lipid peroxides. GSH content increased by 27.47% compared with CK. After rehydration, GSH levels reverted to control values (mean: 217.59 μg/g), mirroring the trend in antioxidant enzymes. However, despite the activation of a robust antioxidant system comprising SOD, POD, CAT, and GSH in autotetraploid, the accumulation of MDA confirmed the occurrence of oxidative damage. This indicates that stress intensity may exceed the full scavenging capacity of the antioxidant system, thereby leading to apparent oxidative damage to the membrane systems.

### 3.5. Transcriptomic Analysis of Autotetraploid Under Drought Stress

**Figure 4:**
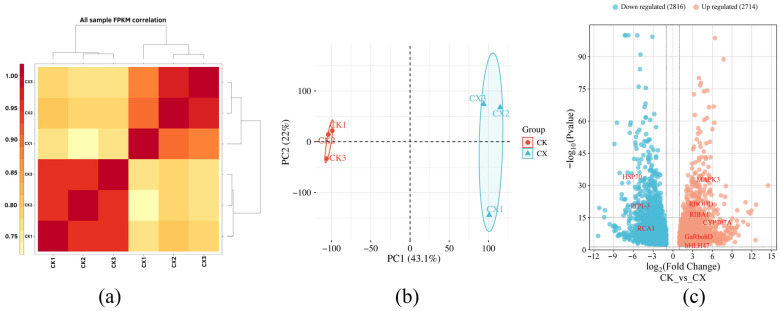
Quality assessment of transcriptome sequencing libraries and screening of DEGs. (**a**) Heatmap of sample correlation analysis; (**b**) Principal Component Analysis (PCA) based on gene expression levels. Red symbols represent the control group (CK), and blue symbols represent the drought stress group (CX) across three biological replicates; (**c**) volcano plot of DEGs (CK vs CX). Red and blue dots represent significantly up-regulated and down-regulated genes, respectively (|log2 FC| ≥ 2, FDR < 0.01).

RNA-seq analysis was performed to investigate drought-responsive genes in the autotetraploid. The GC content ranged from 44.13% to 44.57%, with Q20 and Q30 base percentages exceeding 97.58% and 92.81% respectively. The clean reads were mapped to the *G. arboreum* reference genome, achieving alignment rates from 89.76% to 92.76%. Using FPKM correlation heatmaps, the intra-group correlations for the control group samples were all higher than 0.95, while those for the stress group samples were all higher than 0.88; meanwhile, the inter-group correlations were all greater than 0.74. This clearly demonstrates a high degree of intra-group consistency and significant inter-group differences (Figure 4a). Principal Component Analysis (PCA) revealed significant divergence in gene expression profiles between CK and CX, with PC1 and PC2 explaining 43.1% and 22% of the total variance respectively (Figure 4b). The CK and CX groups were clearly separated in the principal component space, indicating that drought stress induced significant differences in gene expression. Collectively, these findings indicate low sequencing error rates, high data quality, and strong reliability, making the data suitable for subsequent bioinformatics analysis. Using |log2FC| ≥ 2 and FDR < 0.01 as screening criteria for DEGs, 5530 DEGs were identified, including 2714 DEGs significantly up-regulated and 2816 DEGs significantly down-regulated (Figure 4c). These findings further confirm substantial gene expression changes in autotetraploids.

### 3.6. Functional Categorization Analysis of DEGs

**Figure 5:**
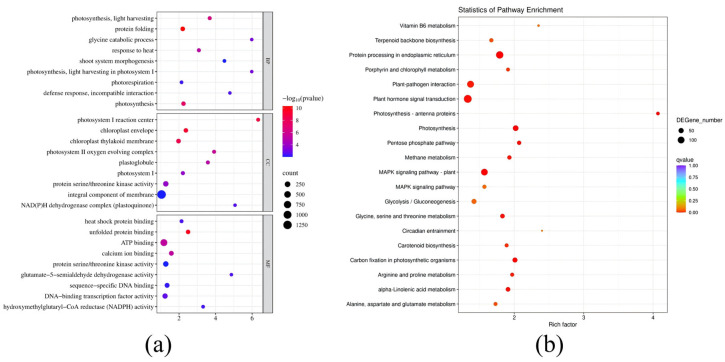
GO and KEGG enrichment analysis of DEGs in the CK vs. CX comparison. (**a**) GO enrichment analysis. The top 9 most significantly enriched GO terms in each category are displayed. BP; CC; MF. The size of the dots represents the number of genes, and the color gradient from red to blue indicates increasing *p*-values (decreasing significance); (**b**) KEGG pathway enrichment analysis. The size of the dots represents the number of genes, and the color gradient from blue to red indicates increasing q value (decreasing significance).

To further elucidate the molecular mechanisms underlying drought stress resistance in autotetraploid, GO functional annotation and KEGG enrichment analysis were performed on all identified DEGs. Based on *p*-value ranking, the top 27 most significantly enriched GO terms are presented in Figure 5a. In the BP category, the most significantly enriched terms included photosynthesis, light harvesting, protein folding, glycine catabolic process, response to heat, and photosynthesis. In the CC category, significant enrichment was primarily observed in the chloroplast envelope, chloroplast thylakoid membrane, protein serine/threonine kinase activity, and integral component of the membrane. In the MF category, the most significantly enriched terms included heat shock protein binding, unfolded protein binding, ATP binding, hydroxymethylglutaryl-CoA reductase (NADPH) activity and DNA-binding transcription factor activity.

The KEGG enrichment analysis results (Figure 5b) indicated that the DEGs were annotated to 120 pathways. The DEGs in autotetraploid were primarily enriched in the following pathways: protein processing in endoplasmic reticulum, MAPK signaling pathway-plant, plant hormone signal transduction, photosynthesis, carbon fixation in photosynthetic organisms and plant-pathogen interaction. The analysis of GO and KEGG enrichment results suggests that under drought stress, autotetraploids adapt to adverse conditions by synergistically regulating photosynthetic apparatus stability, energy metabolism, protein homeostasis maintenance, and multiple stress signal transduction pathways.

### 3.7. Transcription Factors (TFs) Identified by DEGs and PPI Network Analysis

Based on the prediction and classification of TFs within the DEGs, a total of 3079 TFs spanning 79 families were identified. Among these, the *AP2*/*ERF* (171 members), *NAC* (161 members), and *bHLH* (141 members) families exhibited the highest abundance. In contrast, families such as *BSD* and *VOZ* contained only a single TF member.

To further investigate the hub genes governing drought stress responses in autotetraploid, we first constructed the PPI network using screened DEG using Cytoscape version 3.10.4. The network exhibited typical scale-free characteristics. A core functional module was identified, anchored by the molecular chaperone HSP90-6 and the ribosomal protein RPL40B as key nodes. Notably, HSP90-6 served as the central hub, interacting directly with over 45% of the network’s nodes. Topological analysis further revealed that HSP90-6 possessed high betweenness centrality. This network module demonstrated significant functional synergy. Genes directly interacting with HSP90-6 included the ribosomal proteins RPL40B and FKBP17-1. Furthermore, a very strong connection strength (combined weight = 0.883) was observed between the deubiquitinating enzyme BRCC36A and RPL40B, implying a tight coupling between protein stability regulation and stress signal transduction. It is worth noting that robust co-expression relationships were observed among ribosomal proteins; for instance, the connection weight between RPL24A and RPL40B reached 0.9. Further analysis revealed that HSP90-6 maintained direct connections with multiple stress-related proteins, including ABCB19 (weight = 0.444) and CYP57 (weight = 0.429), highlighting its central status as a hub node for integrating diverse stress signals. The PPI network revealed potential regulatory modules, providing candidate targets for subsequent functional validation.

### 3.8. Analysis of Key Genes Responding to Drought Stress

By focusing on highly significant pathways identified by KEGG and GO enrichment analyses, and integrating key modules such as Pfam annotations, NR annotations, and Swiss-Prot, a total of 34 membrane-associated DEGs were identified (20 up-regulated, 14 down-regulated) (Figure S2a). Fifteen genes annotated as membrane receptor kinases, including LRR receptor-like kinases (ERL1, HPCA1-like) and cysteine-rich receptor-like kinases (CRK3), showed significantly up-regulated expression. The calcium/calmodulin-regulated receptor-like kinase CRLK1 (*Cotton_A_21680*) also showed strong induction, indicating these proteins play a crucial role in perceiving and transducing drought signals. Furthermore, multiple members of the ABC transporter family exhibited differential expression. ABCG11 (*Cotton_A_36971*), ABCG3 (*Cotton_A_27428*), ABCA2 (*Cotton_A_09211*), and ABCD1 (*Cotton_A_26725*) were generally up-regulated, while members such as ABCC3 were suppressed. This subfamily is known to participate in the transmembrane transport of lipid precursors such as waxes and cutin. Its enhanced expression may be associated with direct drought resistance mechanisms involving leaf cuticle strengthening and non-stomatal water loss [24,25,26]. Concurrently, the aquaporin PIP1-3 (*Cotton_A_25965*) was down-regulated. In comparison, the plasma membrane Ca^2+^-ATPase ACA9 (*Cotton_A_31403*) was up-regulated. This suggests that cells may regulate stress-related calcium signaling by enhancing calcium efflux, which likely contributes to maintaining membrane potential stability.

Down-regulation of HSP70 (*Cotton_A_00363*) and Rubisco activase RCA1 (*Cotton_A_22968*) was observed, while up-regulation occurred in the ATP-dependent Clp protease ATP-binding subunit (ClpX) and small subunit RIBA1 (*Cotton_A_24804*). The up-regulation of the stress-induced transcription factor bHLH47 (*Cotton_A_09219*) and changes in the expression of JAZ6 (*Cotton_A_01448*) further suggest that these genes may be involved in regulating drought tolerance in autotetraploids.

Most genes in the “heat response” category showed significantly reduced expression under drought stress, primarily involving protein folding and processing as well as stress response functions (Figure S2b). Among these, the respiratory burst oxidase homologs RBOHD (*Cotton_A_13752*) and GaRbohD (*Cotton_A_18772*) were up-regulated, while several DnaJ protein homologs (*Cotton_A_01704*, *Cotton_A_27260*, *Cotton_A_15800*, *Cotton_A_22333*, and *Cotton_A_36806*), ClpB (*Cotton_A_18300*, *Cotton_A_14703*, and *Cotton_A_33967*), and a lipid transporter (*Cotton_A_07240*) were down-regulated. Meanwhile, the peptide prolyl cis-trans isomerase FKBP62 modulates ROS-related signal transduction efficiency by regulating conformational changes in signaling proteins (such as kinases).

This study also revealed that multiple components of the MAPK cascade were significantly regulated, including MAPK3 (*Cotton_A_02201*), MAPK7 (*Cotton_A_02010*), MAPK homolog MMK1 (*Cotton_A_23784*), and its upstream MAPKK6 (*Cotton_A_09342*), which were up-regulated (Figure S2c). These kinases are part of a complex phosphorylation network that amplifies and transmits signals from membrane receptors, serving as critical components in plant responses to abiotic stress [27,28]. Additionally, *WRKY* family members (e.g., *WRKY22*, *WRKY26*, *WRKY33*) were significantly up-regulated.

### 3.9. Validation of DEGs by qRT-PCR

**Figure 6:**
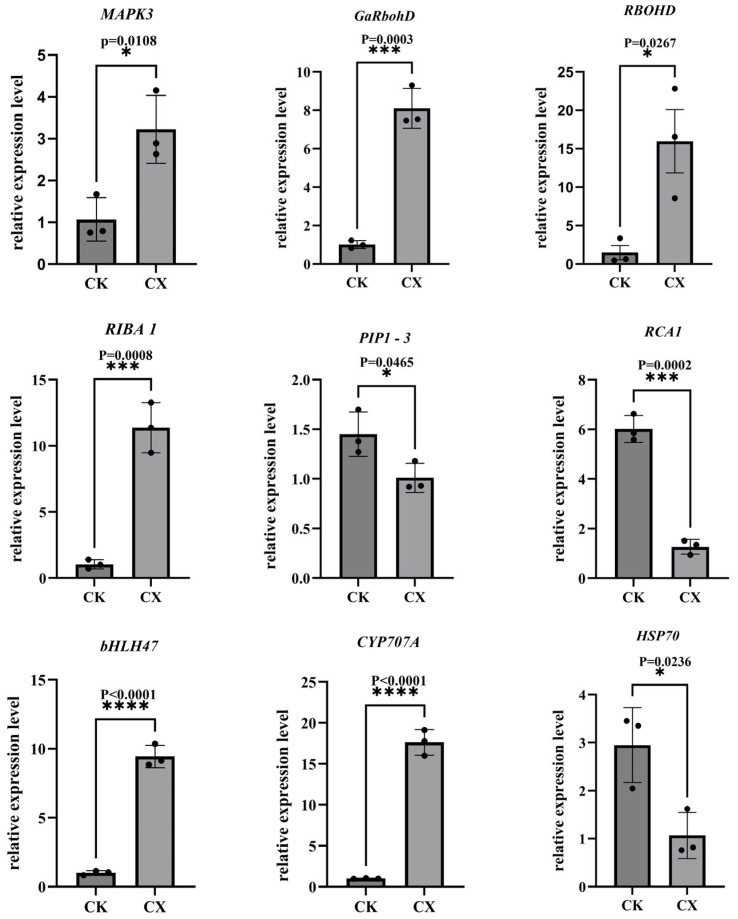
Validation of gene expression by qRT-PCR. The asterisk (*) indicates a significant difference in gene expression between the control and experimental groups, *** represents a significant difference among the different treatments in the same varieties at *p* < 0.001, **** represents a significant difference among the different treatments in the same varieties at *p* < 0.0001.

The reliability of RNA-Seq data was verified by qRT-PCR using nine randomly selected DEGs. The transcript profiles of these selected genes in the qRT-PCR analysis showed a similar pattern as being identified by the FPKM from RNA-seq under corresponding treatments (Figure 6). These results confirm the reliability of RNA-Seq data.

## 4. Discussion

### 4.1. Physiological Responses of G. herbaceum Autotetraploids Under Drought Stress and Their Potential for Breeding Applications

Polyploidization is a major driver of plant evolution and speciation, playing a crucial role in plant adaptation to environmental stress. Chromosome doubling results in an increased copy number of antioxidant enzyme genes, thereby enhancing ROS-scavenging capacity through gene-dosage effects [29,30]. The autotetraploid materials used in this study exhibited a high capacity to maintain photosynthetic parameters, accumulate osmoregulatory substances, and maintain antioxidant enzyme activity under drought stress. The other results indicated that stomatal limitation primarily governs the initial phase of photosynthetic suppression [31,32]. Following rehydration, the recovery rate of *Pn* significantly exceeded that of *Gs*, suggesting that non-stomatal factors (involving enhanced Rubisco function or related photosynthetic enzyme systems) play a crucial role in enhancing carbon assimilation efficiency [33]. This reflects the resilience of photosynthetic mechanisms in autotetraploid plants during stress recovery, a phenomenon consistent with the general characteristics of polyploid plants [34,35,36].

In the progeny of an autotetraploid, the S2 generation exhibited greater stability than the S1 generation. Specifically, the proportion of normal tetrads increased to 85.17%, and the proportion of normal pollen grains reached 87.33%. These findings indicate that meiotic processes in the polyploids are gradually normalizing, laying a foundation for the stable inheritance of drought resistance traits [37]. Concurrently, we selected the S4 generation of artificial autotetraploid as the experimental material, and the reason is that the chromosome composition, cytological behavior, and phenotypic traits of this generation have become essentially stable after four consecutive self-pollination inbreeding cycles, and the genetic background is uniform, with significantly reduced levels of aneuploidy and mosaicism, effectively ensuring the reproducibility and reliability of experimental results. This autotetraploid material serves as a breeding intermediate or germplasm resource. Its unique genomic composition and phenotypic characteristics provide a valuable experimental platform and genetic material for subsequent polyploid breeding, gene function validation, and improvement of important traits.

### 4.2. Physiology Change in Autotetraploids Under a Gradual Water Deficit

Drought represents a major abiotic stress limiting cotton productivity, exerting significant impacts on the photosynthetic system and metabolic homeostasis [38,39]. Previous studies have confirmed that the accumulation of Pro and SS plays a central role in drought adaptation by maintaining cellular water potential and protecting macromolecular structures [40,41]. Dong et al. [42] found that birch trees overexpressing *BpWRKY53* exhibited enhanced drought tolerance through increased proline accumulation and antioxidant enzyme activity. Consistent with these studies, the autotetraploid material in this study accumulated higher levels of proline and soluble sugars during the peak stress period. Antioxidant enzyme activities increase to scavenge excess ROS induced by drought, while non-enzymatic antioxidants collectively maintain membrane integrity [43,44,45]. In this study, autotetraploid plants maintained higher antioxidant enzyme activity under stress conditions, consistent with the widely held view that polyploid plants generally exhibit stronger antioxidant capacity. However, it warrants further investigation whether this enhancement represents a simple “dose effect” or reflects “systemic reorganization” at the network level.

### 4.3. Transcriptional Reprogramming Underlies Drought Resistance

Polyploidy complicates transcriptional regulation and increases phenotypic diversity in organisms [46,47]. The MAPK pathway is co-enriched with plant hormone signal transduction (e.g., ABA, ethylene, JA) and Ca^2+^ signaling pathways, forming a multi-dimensional regulatory axis [48]. Previous studies have shown that *GhMAPK3* is primarily expressed in roots and leaves, and its inhibition exerts a significant negative impact on salt and drought tolerance in *G. hirsutum* [49]. Chen et al. [50] proposed that the MAPK cascade pathway GhMAP3K62-GhMKK16-GhMPK32 plays a role in cotton’s drought response through ABA-dependent stomatal movement. Polyploidization may lead to the functional divergence of MAPK pathway members. For instance, *GhCIPK6* homoeologs in *G. hirsutum* have evolved into sub-functionalized modules that positively and negatively regulate drought resistance, further enriching the precision of signal regulation [51].

In PPI network analysis (based on the STRING database and Cytoscape) and plant hormone signaling pathways, HSP70 and HSP90 were predicted to be candidate hub nodes in the protein–protein interaction network. As core components of molecular chaperones, HSP70 and HSP90 significantly enhance the capacity for cellular homeostasis recovery under drought stress by maintaining correct protein folding and preventing toxic aggregation. While most HSP70s are typically up-regulated in response to biotic stress and participate in PTI and ETI responses [52], we observed a down-regulation of HSP70 in this study. This is likely an active, programmed adaptive response, reflecting a strategic shift in cotton from “growth maintenance” to “survival assurance” amidst the “energy crisis” induced by drought. Tsering et al. [53] provided the first systematic revelation of the mechanism by which the HSP90 molecular chaperone specifically stabilizes ABCB-type auxin transporters in plants via the co-chaperone TWD1. Consistent with this finding, transcriptomic analysis in this study identified HSP90, multiple members of the ABC transporter family, and FKBP62 as potential interacting factors through PPI network predictions. These findings provide valuable insights into the complexity of the plant HSP90 regulatory network.

## 5. Conclusions

This study analyzed the physiological and transcriptional responses of an autotetraploid line of *G. herbaceum* under drought stress. The line reduced water loss through stomatal-limited photosynthetic inhibition; following rehydration, non-stomatal factors contributed to a partial recovery of photosynthesis, while osmotic regulators and antioxidant enzyme activities increased significantly. Transcriptome analysis revealed enrichment in MAPK signaling, heat response, hormone signaling, and membrane-related pathways, identifying nine candidate genes (*RBOHD*, *CYP707A*, *RIBA1*, *bHLH47*, *MAPK3*, *GaRbohD*, *RCA1*, *HSP70*, *PIP1-3*) and interacting factors (HSP90, ABC transporters, FKBP62). Due to the lack of diploid controls, the above conclusions are limited to this material itself and do not involve comparisons of polyploid advantages.

It should be noted that this experiment did not include a diploid ancestral control directly corresponding to this autotetraploid. Our findings describe only the response patterns of this specific autotetraploid material under drought stress. Future studies must include paired diploid controls and conduct direct comparisons under identical conditions to verify whether polyploidization confers a drought-tolerance advantage.

## Figures and Tables

**Table 1:** Primer sequences for the qRT-PCR reaction.

Gene ID	Gene Name	Primer Sequences (5′-3′)	
		F	R
*nbis-gene-29687*	*MAPK3*	GATTCCTCCGCCTTTAAGG	AATACTGGCAATGCTCCTCA
*nbis-gene-36157*	*GaRbohB*	CACCAGCGTTTACGAGGAA	GGCTTGGCGAAGTGAGATT
*nbis-gene-8314*	*RBOHD*	CTGAAACCTACTCAAGAGAACAACC	AAGCCACAGCATCATAACCC
*nbis-gene-1760*	*RIBA1*	GGTTTTGCTTCCATCCTTG	CCTTCATTCTCTCTGTCTTCATC
*nbis-gene-37784*	*PIP1-3*	GGGATTCCAAGGAGATAACC	GCATCAGTAGCAGAGAAAACAGT
*nbis-gene-24066*	*RCA1*	GGTTTCTACATCGCTCCTGC	TTGACCTTTGCCTCCCCA
*nbis-gene-25356*	*bHLH47*	CGAAGACGAAGGGAAAGGTT	TTTGCCAGGTCAAGGAAGAG
*nbis-gene-18385*	*CYP707A*	TGAAAGCAAGGAAAGAGATAGC	AATTTGTTCGTCGGTGAGG
*nbis-gene-23992*	*HSP70*	CAACCATAACCCGAGCCA	ACCAACGAGGACGACATCA
Internal Reference	*GhUBQF*	GAATGTGGCGCCGGGACCTTC	ACTCAATCCCCACCAGCCTTCTGG

## Data Availability

The raw data supporting the conclusions of this article will be made available by the authors on request.

## Supplementary Materials

The following supporting information can be downloaded at: https://www.mdpi.com/article/10.3390/genes17040470/s1, Figure S1: Gene co-expression interaction network underlying drought stress response. Figure S2: Expression profiles of genes comprising key functional modules in drought-stressed *G. herbaceum* autotetraploid.

## Abbreviations

The following abbreviations are used in this manuscript:

DEGs	Differentially expressed genes
TF	Transcription factor
CK	The control group
SRWC	Soil relative water content
CX	The drought stress group
RW	Re-watering group
SPAD	Relative chlorophyll content
Pn	Net photosynthetic rate
Ci	Intercellular CO_2_ concentration
Tr	Transpiration rate
Gs	Stomatal conductance
SOD	Superoxide dismutase
POD	Peroxidase
CAT	Catalase
MDA	Malondialdehyde
TBA	Thiobarbituric acid
Pro	Proline
SS	Soluble sugar content
GSH	Glutathione
RIA	RNA integrity number
BP	Biological processes
CC	Cellular components
MF	Molecular functions
