# Supplementary material for: Integrated Insights into Drought Tolerance Mechanism of the Autotetraploid from Gossypium herbaceum by Transcriptome and Physiological Analyses
Source: Genes (Basel). 2026 Apr 17;17(4):470. doi: 10.3390/genes17040470 (PMC13115804; doi:10.3390/genes17040470)
Supplement: Supplementary file 1 [file genes-17-00470-s001.zip › genes-4248060-supplementary.pdf]

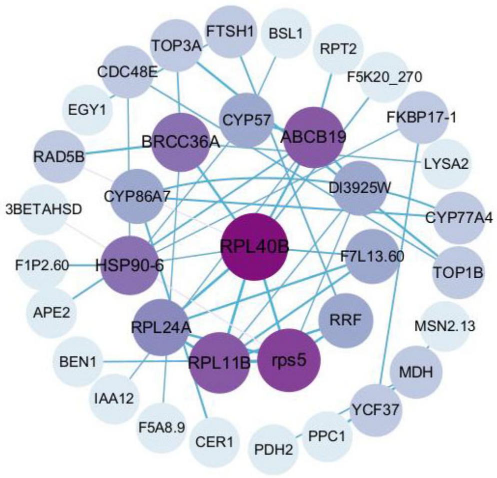

Figure S1. Gene co-expression interaction network underlying drought stress response. This network was constructed based on co-expression relationships among differentially expressed genes under drought stress and visualized using Cytoscape. The network exhibits a characteristic scale-free topology. Nodes represent genes, with their sizes proportional to degree centrality and color intensity reflecting betweenness centrality. Edges denote significant co-expression relationships between gene pairs (absolute correlation coefficient > 0.85).

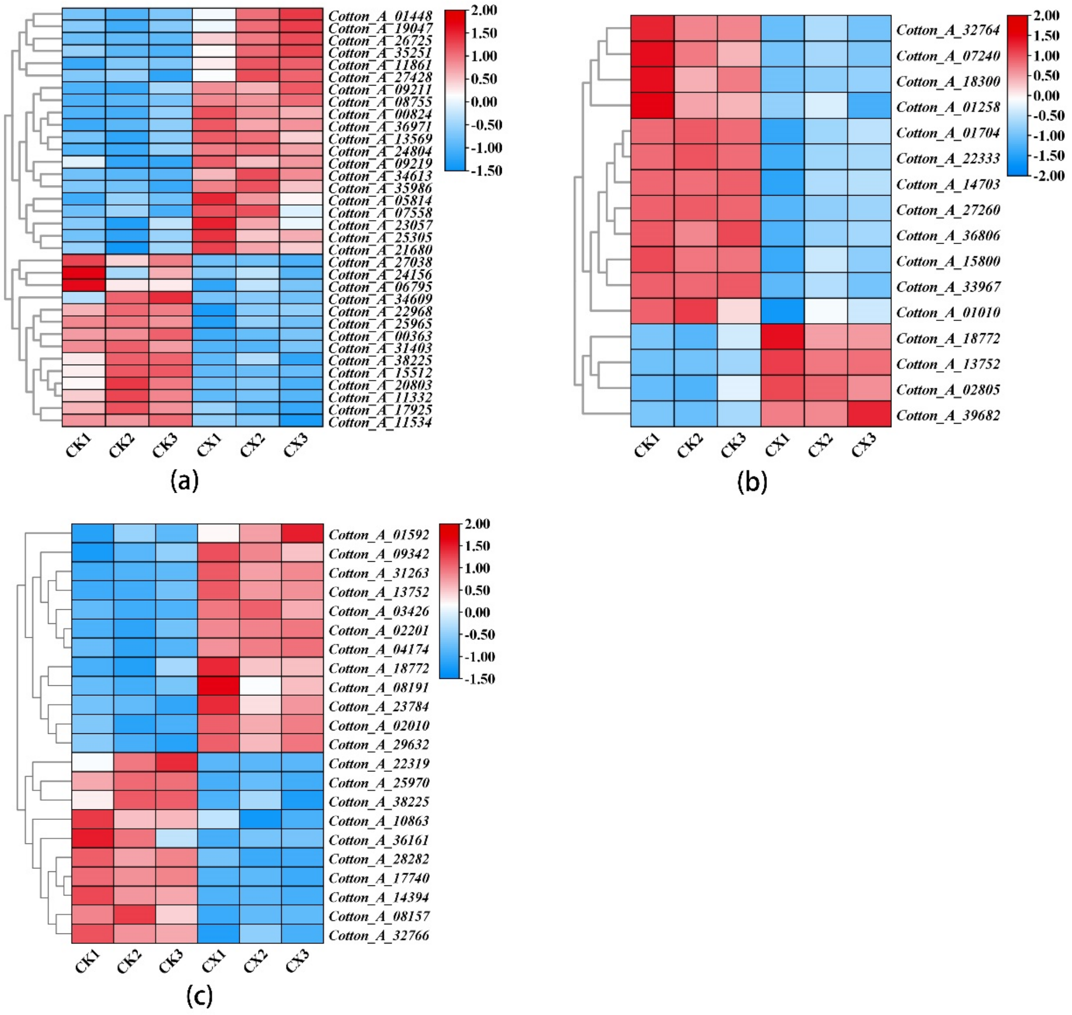

Figure S2. Expression profiles of genes comprising key functional modules in drought-stressed *G. herbaceum* autotetraploid. (**a-c**) Heatmaps display the expression patterns of differentially expressed genes belonging to three core functional modules — plant hormone signal transduction and biomembrane, response to heat, and MAPK signaling pathway – plant—in control versus drought-stressed groups. Samples include three biological replicates each of the control (CK1-CK3) and drought-treated (CX1-CX3) groups. Each row represents a gene, and each column represents a sample. Expression levels are Z-score normalized and depicted by a color gradient: red indicates high expression, and blue indicates low expression. Both genes and samples were clustered hierarchically using Euclidean distance.
